# Rhodium Nanoparticle-Supported Graphitic Carbon-Encapsulated Nickel Metal Core Electrocatalyst via Pulsed Laser Ablation for Hydrogen Evolution Reaction

**DOI:** 10.1021/acsami.5c07778

**Published:** 2025-07-29

**Authors:** Yewon Oh, B. N. Vamsi Krishna, Hyeon Jin Jung, Anju Toor, Seung Jun Lee

**Affiliations:** † Department of IT and Energy Convergence (Brain Korea 21 FOUR), 34934 Korea National University of Transportation, Chungju 27469, South Korea; ‡ School of Materials Science and Engineering, 1372 Georgia Institute of Technology, Atlanta, Georgia 30332, United States; § Nano Convergence Materials Center, Emerging Materials R&D Division, 87797 Korea Institute of Ceramic Engineering and Technology (KICET), Jinju 52851, South Korea

**Keywords:** Pulsed laser ablation and irradiation, hydrogen evolution reaction, Rh−Ni@Graphitic carbon layer, Nickel metal core structure, Rhodium nanoparticles, graphitic carbon layer

## Abstract

The hydrogen evolution reaction (HER) in acidic media exhibits high reaction rates but is often hindered by stability challenges under corrosive conditions. In this study, we introduce a novel approach to synthesizing nickel nanoparticles encapsulated in nitrogen-doped carbon layers decorated with noble metals (Ir and Rh), with the aim of improving catalytic activity, durability, and conductivity for HER applications. Using a two-step pulsed laser ablation and irradiation process, this environmentally friendly synthesis facilitates the rapid production of Rh- and Ir-decorated Ni@GC composites with robust metal–support interactions. The resulting catalysts exhibit outstanding HER performance in 0.5 M H_2_SO_4_ acidic electrolyte, achieving a current density of 46 mV at 10 mA cm^–2^ with a low Tafel slope of 36 mV dec^–1^. The optimized Rh-Ni@GC electrocatalyst showed long stability results over 24 h using a chronoamperometry test. This work demonstrates a sustainable and effective method for developing high-performance electrocatalysts for hydrogen production.

## Graphical abstract

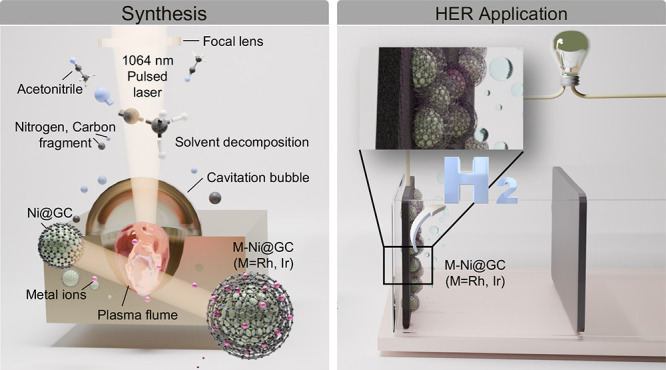

## 1 Introduction

Hydrogen (H_2_) has emerged as a promising renewable and clean energy source, offering a sustainable alternative to fossil fuels, such as coal and oil. Its importance continues to grow due to its potential to address the global demand for environmentally friendly energy solutions. Various methods exist for hydrogen production, including steam reforming, photoelectrochemical hydrogen generation, water thermolysis, and water electrolysis.^1,2^ Among these, water electrolysis has received significant attention due to its ability to produce high-purity hydrogen reliably. The hydrogen evolution reaction (HER) under acidic conditions delivers high efficiency, avoiding the slow water dissociation steps required under alkaline conditions. However, the aggressive acidic environment poses significant corrosion challenges for catalysts. During the electrocatalytic reaction, the electrocatalyst undergoes degradation, which leads to a decline in both its activity and its stability. Achieving long-term stability is therefore critical for the sustainable operation of energy devices.

Platinum-based catalysts are widely used as an efficient catalyst for HER due to their better activity, especially in acidic electrolytes.^3^ However, its high cost has driven extensive research into catalysts with lower loading of noble metal and non-noble metal catalysts as cost-effective alternatives.^4,5^ These alternatives often fall short in overpotential performance or stability issues under operational conditions. In addition to economic concerns, surface oxidation poses a significant challenge in developing cathode materials, as it leads to the loss of active sites, structural degradation, reduced electrical conductivity, increased overpotential values, and formation of passivation layers. To address the above issues, the graphitic carbon layer serves as a crucial protective barrier, offering both physical and chemical defense to effectively prevent surface oxidation and agglomeration.^6,7^ This protective mechanism is essential for maintaining the catalyst’s activity and stability under harsh conditions. Recently, the integration of protective layers, such as N-doped graphitic carbon with minimal noble metal loadings, has gained profound interest. It presents a promising strategy to mitigate degradation while ensuring high catalytic performance and cost-effectiveness. Previous studies have demonstrated that Rh and Ir nanoparticles show corrosion resistance and intrinsic activity, while graphitic carbon layers provide structural stability and electronic conductivity.^8,9^ And the use of nickel-based core structures offers a cost advantage and great synergistic catalytic activity.

Despite these advancements, the clean and scalable synthesis of such integrated structures remains limited. Pulsed laser ablation in liquid (PLAL) is a versatile and environmentally friendly technique for synthesizing nanoparticles (NPs).^10^ It has gained significant attention for producing catalysts, including those for HER. This method enables the rapid synthesis of materials under ambient conditions without involving complex processing steps or byproduct formation.^11^ The PLAL method with its modifications has proved to be attractive as a simple and easy-to-use method that allows for preparation of diverse unique nanostructures at the laboratory scale, which is often difficult or impossible to synthesize by other approaches.^9,12^ Through ablation of solid targets immersed in a liquid medium, or irradiation of powders dispersed in liquid, PLAL has demonstrated a high potential in preparing nanomaterials with different morphology, size distribution, chemical composition, and surface defects, and even with metastable phases that are difficult to achieve via more conventional wet-chemistry approaches.^13,14^ Furthermore, the absence of hazardous chemicals and reducing agents minimizes the risk of groundwater contamination.^15^ The exclusion of unwanted chemicals facilitates the synthesis of catalysts with clean surfaces, effectively preventing the adsorption of impurities and ensuring readily accessible active sites, thereby enhancing catalytic activity.^16^ In contrast to conventional wet-chemistry methods, the laser synthesis method demonstrated in this work drastically reduces processing time to 30 min, providing a more energy-efficient and scalable approach. These improvements collectively highlight the potential of a minimal noble metal-decorated Ni@GC catalyst to achieve high performance with lower costs and simplified synthesis, addressing key challenges in electrocatalyst development.

In this study, minimal loading of noble metal-decorated Ni@GC was synthesized using an innovative two-step pulsed laser technique. First, the PLAL method was employed to synthesize N-doped graphite carbon-encapsulated nickel nanoparticles in a one-pot process at room temperature. Subsequently, Ir and Rh nanoparticles were decorated onto Ni@GC via PLAL, a technique that facilitates strong binding between the NPs and the Ni@GC surface. This structure provides numerous active sites, improved electrical conductivity, and robust electronic interactions, collectively enhancing the catalytic efficiency and stability. SEM-EDS and TEM analyses were conducted to examine the morphology and elemental distribution of the synthesized materials. SEM-EDS confirmed the homogeneous dispersion of noble metal particles, and high-resolution TEM images showed nanoscale features and structural integrity favorable to catalytic activity. Additionally, XPS measurements were employed to analyze the surface composition and oxidation states of the elements, providing deeper insights into the electronic structure and potential active sites. The synthesized catalyst was successfully applied to sustainable hydrogen production in acidic media, demonstrating its potential as an efficient and durable solution for hydrogen energy technologies.

## 2 Materials and Methods

### 2.1 Materials

Ni metal plate (99.98% trace metals basis), rhodium­(III) nitrate solution (Rh­(NO_3_)_3_; ∼10% (w/w) (Rh in >5 wt % HNO_3_)), and iridium­(III) chloride hydrate (IrCl_3_·*x*H_2_O; 99.9%) were purchased from Sigma-Aldrich, USA. Acetonitrile (CH_3_CN; ≥99.5%) and ethyl alcohol (C_2_H_6_O; ≥99.5%) were purchased from Daejung Chemicals, South Korea. Isopropyl alcohol ((CH_3_)_2_CHOH; extra pure) was purchased from Samchun Chemicals, South Korea. All chemicals and reagents were used directly after purchase.

### 2.2 Synthesis of Graphitic Carbon (GC)-Encapsulated Nickel (Ni) Nanospheres (Ni@GC)

Carbon-encapsulated Ni nanospheres were synthesized via the pulsed laser irradiation in liquid (PLIL) process. A Ni plate was immersed in 10 mL of acetonitrile and ablated with focused Nd:YAG laser (focal length = 30 mm, frequency = 10 Hz, pulse width = 7 ns, fundamental wavelength = 1064 nm, power = 100 mJ/pulse) for 30 min. The resulting colloidal solution was centrifuged several times with absolute ethanol and dried overnight. The synthesized Ni nanospheres are referred to as Ni@GC.

### 2.3 Synthesis of Noble Metal-Decorated Ni@GC

Ni@GC material decorated with noble metal nanoparticles was synthesized by laser irradiation after adding Ir and Rh precursors to a colloidal solution of presynthesized Ni@GC. Ten μM aqueous Ir precursors were added to the prepared colloidal Ni@GC solution, and a nonfocused 90 mJ/pulsed laser beam was irradiated for 10 min with continuous 300 rpm of stirring. The resulting powder of Ir nanoparticles decorated Ni@GC was referred to as Ir-Ni@GC. The same procedure was used for the Rh-based sample, referred to as Rh-Ni@GC. The following weight ratios for the Ir-based sample are 0.1, 0.15, 0.2, and 0.25 mM, which are named Ir-Ni@GC-1, Ir-Ni@GC-2, Ir-Ni@GC-3, and Ir-Ni@GC-4, respectively. The respective weight ratios for the Rh-based sample are 0.5, 0.75, 1, and 1.25 mM rhodium nitrate solution, which are named Rh-Ni@GC-1, Rh-Ni@GC-2, Rh-Ni@GC-3, and Rh-Ni@GC-4, respectively.

### 2.4 Formation of Noble Metal-Decorated Graphitic Carbon Coated Ni Nanospheres via Pulsed Laser Technique

Figure 1(a) illustrates the two-stage synthesis process of noble metal-decorated, carbon-encapsulated nickel nanospheres through pulsed laser ablation and irradiation in liquid. In step 1, a focused 1064 nm pulsed laser beam was irradiated onto the nickel metal target, which is immersed in acetonitrile. Under the high-energy pulsed laser beam, the metal target surface interacts with it, forming a high-temperature (∼2000 K) and high-pressure (∼100 atm) plasma plume, leading to the immediate ionization/atomization of the metal. During the ablation process, the organic solvent (acetonitrile) decomposes, generating free carbon atoms and clusters as well as hydrogen and oxygen radicals and gases. During the continuous irradiation process, the Ni nanospheres were formed from the source of the Ni plate and the shell structure formed on the surface of Ni species from acetonitrile decomposition. Then, the graphitic carbon shell-coated Ni nanosphere (Ni@GC) material was formed. In the second stage, the irradiation of Ni@GC suspension with noble metal precursors leads to successive reduction of metal species to the respective metal nanoparticles with simultaneous decoration on the surfaces of Ni nanospheres. This successful reduction of metal species to metal nanoparticles is due to the synergetic effect of the laser beam and radicals produced in the solvent. The procured powders are then termed Ir-Ni@GC and Rh-Ni@GC materials. The pulsed laser irradiation process effectively establishes strong metal–support interactions of Ir NPs and Rh NPs with Ni@GC spheres.

**Figure 1:**
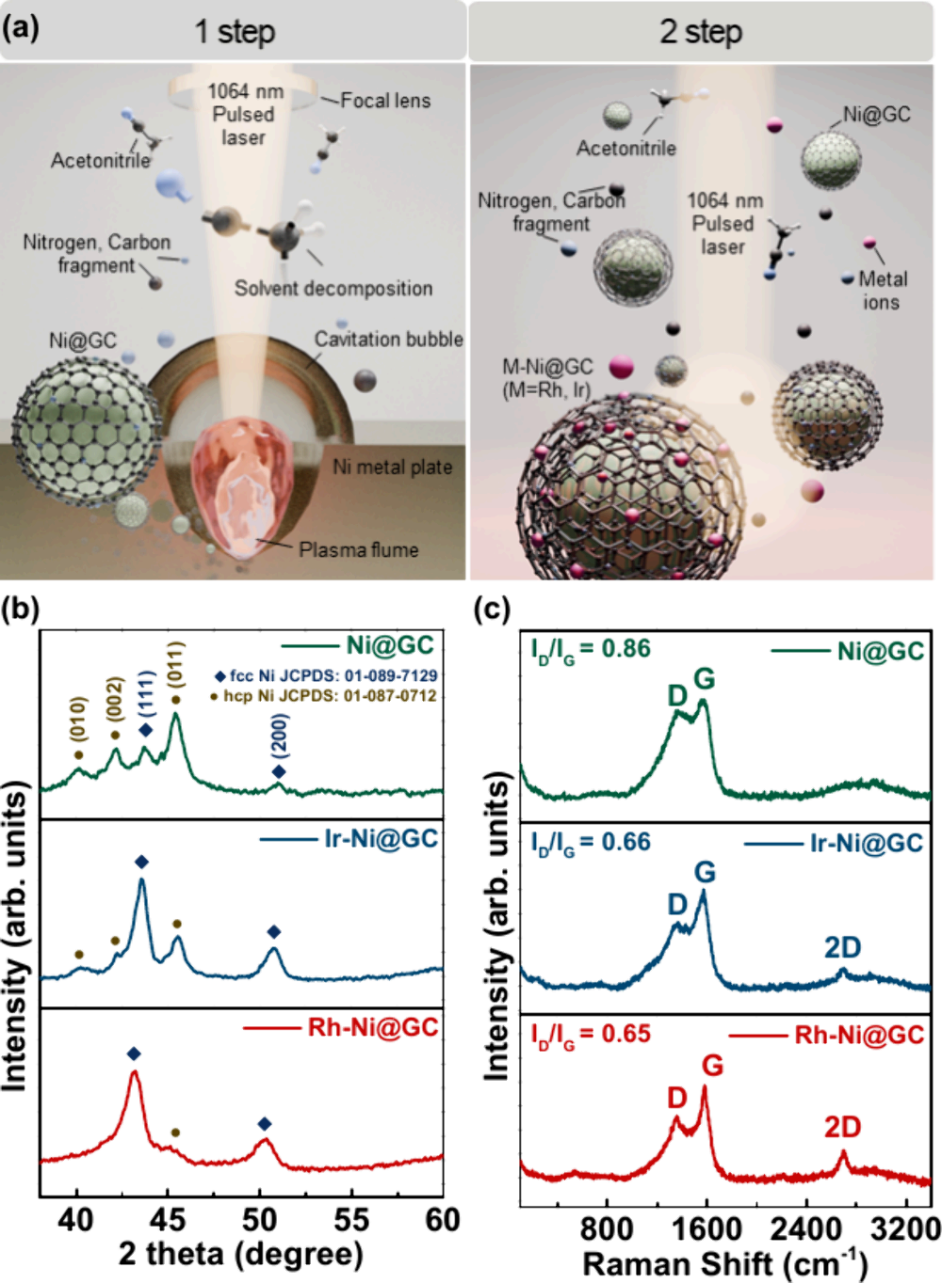
(a) Schematic illustration of the two-step synthesis process. (b) XRD and (c) Raman spectra for Ni@GC, Ir-Ni@GC, and Rh-Ni@GC materials.

### 2.5 Preparation of Electrode Materials

Electrochemical investigations were carried out using a three-electrode configuration, with a Pt wire as the counter electrode, a Ag/AgCl electrode as the reference electrode, and a glassy carbon electrode (GCE, ∼0.07 cm^2^ area, 3 mm diameter) coated with the synthesized Rh-Ni@GC, Ir-Ni@GC, or Ni@GC materials as the working electrode. The mass loading of the materials was about 0.02 mg (282.9 μg/cm^2^) used in the total volume of the ink (250 μL). The working electrode (WE) ink was prepared by dispersing approximately 1.0 mg of Rh-Ni@GC, Ir-Ni@GC, or Ni@GC in a solvent mixture of ethanol and water (1:1, v/v) with 10 μL of a Nafion solution as a binder. The dispersion was subjected to ultrasonication for 30 min to produce the electrocatalyst ink. Then, 5 μL of the ink was drop-cast onto the surface of the glassy carbon electrode (GCE), achieving a mass loading of 0.019 mg (282 μg/cm^2^).

## 3 Results and Discussion

The phase structures of prepared Ni@GC, Ir-Ni@GC, and Rh-Ni@GC samples were investigated by X-ray diffraction (XRD) and the results are shown in Figure 1(b). The XRD results confirmed the mixed phase of face-centered cubic/hexagonal close-packed (fcc/hcp) Ni nanospheres. The observed characteristic peaks at 40.4°, 42.3°, and 45.6° correspond to hcp (JCPDS card no. 01-089-7129) and the characteristic peaks observed at 44.4° and 51.8° correspond to fcc (JCPDS card no. 01-087-0712) as shown in Figure 1(b). The obtained XRD results are well in agreement with the previously published literature.^17^ Due to the low specific heat of acetonitrile (2.23 J·K^–1^·g^–1^), the liquid environment cools rapidly, enabling the synthesis of a metastable hcp-phase of nickel instead of a more stable fcc-phase nickel phase. During the secondary irradiation process for metal coordination, the thermodynamically stable fcc Ni structure can be observed in the XRD patterns of Ir-Ni@GC and Rh-Ni@GC. Due to the small amount of decorated noble metal in the sample, the diffraction signals of Ir and Rh are too weak to be detected by X-ray diffraction (XRD). As a result, no distinct peaks corresponding to these noble metals were observed in the XRD patterns of the Ir-Ni@GC and Rh-Ni@GC samples. Also, broad peaks are observed for Ir-Ni@GC and Rh-Ni@GC samples, which is due to enhancement in the GC shell thickness on the surface of Ni@GC material, leading to the broadening of diffraction peaks of Ni nanospheres as can be seen in Figure 1(b).^18^ The bond strength of the metal species mostly depends on the d-band center of the metal; the higher the d-band center, the stronger the interactions of the metal substrate.

The d-band center reduces from the left side to the right side in the periodic table of elements such as Ru > Rh > Pd > Ag and Os > Ir > Pt > Au.^19^ Thus, Rh NPs exhibit the strongest interaction with Ni@GC, resulting in a peak shift of ∼0.4° in the (111) crystallographic plane of Ni@GC, while Ir NPs induce a shift of approximately 0.2°. The Raman analysis was employed to gain further insights into the surface chemical composition. Figure 1(c) shows the Raman spectra of the prepared Ni@GC, Ir-Ni@GC, and Rh-Ni@GC samples. The three main characteristic peaks of graphitic carbon, i.e., D, G, and 2D peaks, are observed at 1336, 1570, and 2702 cm^–1^ as shown in Figure 1(c). Since the Ni is silent in the Raman spectrum, no other peaks were identified in the prepared samples.^20^ The calculated peak intensity ratio values of *I*_*D*_ */I*_*G*_ are 0.86, 0.66, and 0.65 for the Ni@GC, Ir-Ni@GC, and Rh-Ni@GC samples, respectively.^21^ The observed difference between the *I*_*D*_ */I*_*G*_ values indicates a change in the number of defects in the graphitic layers. The lower I_D_/I_G_ ratio of the Rh-Ni@GC sample is due to the increase in graphitic carbon, which likely enhances the electrical conductivity.^22^ For instance, the 2D peak shift is observed for the Ni@GC sample when compared to the remaining Ir-Ni@GC and Rh-Ni@GC samples. Such a peak shift in Raman peaks might be related to the electronic structure modifications of graphitic layers obtained from the interaction of the core of Ni nanospheres and the graphitic carbon shell.^23^

The morphological and microstructural properties of the materials were investigated by field emission scanning electron microscopy (FE-SEM) and transmission electron microscopy (TEM) techniques. Figure 2(a) shows the FESEM image of the Ni@GC sample containing numerous irregular nanospheres fully covered with a GC shell. The FESEM images in Figure 2(b and c) confirm that the tiny Ir and Rh nanoparticles are uniformly distributed on the surface of the Ni@GC sample. The good morphological structures and better-connected surface states played a crucial role in the electrocatalytic performances by improving the electron transport properties from the surface of the electrode to the electrolyte. The high-magnification TEM images of the Ni@GC, Ir-Ni@GC, and Rh-Ni@GC samples are shown in Figure 2(d–f), respectively. These HRTEM images demonstrate the uniform growth of the GC shell on the Ni nanosphere surfaces. The *d*-spacing value of 0.19 nm is attributed to the (011) plane of hcp Ni nanospheres as represented in Figure 2(d). From the observed TEM images, noble metal nanoparticles (Ir and Rh) are uniformly decorated on the surface of the Ni@GC material. The calculated *d*-spacings of 0.22 and 0.21 nm are attributed to the (111) plane of Ir and (111) plane of Rh nanoparticles as depicted in Figure 2(e and f), respectively. In addition, from high-magnification TEM images of Figure 2(d, e, and f), the *d*-spacing of 0.34 nm is attributed to the (002) plane of the GC shell. This GC shell was formed from a carbon-rich organic solvent, which decomposed and condensed during the ablation process to coat the surface of the Ni nanospheres. From TEM images, the thicknesses of the GC shells on the Ni nanosphere surface are ∼1.719 nm (Ni@GC), ∼7.903 nm (Ir-Ni@GC), and ∼12.868 nm (Rh-Ni@GC), respectively. The TEM results shown in Figure 2(d–f) are in alignment with the XRD and FESEM results.^24,22^ The energy dispersive spectrometer (EDS) layered image and elemental mappings with elemental weight ratios for Ni@GC, Ir-Ni@GC, and Rh-Ni@GC samples are presented in Figure 2(g–i), respectively. The elemental mapping images demonstrated the homogeneous distribution of elements, which indicated that the Ni nanospheres were encapsulated by the GC shell. Similar weight ratios were identified for both Ir (7.33 wt %) and Rh (7.31 wt %) nanoparticles as shown in Figure 2(h and i), respectively, which were incorporated on the surface of Ni@GC materials. As observed in the FESEM and TEM results, intimate interfacial contact was formed between the composite materials, instead of a physical mixture of these materials. This structural feature is important for the enhanced charge transfer properties. With the GC shell thickness increasing from 1.719 to 12.868 nm, the diffusion coefficients of the oxidation/reduction peaks increase. The reason could be that the GC shell not only enhances the electrical conductivity of the samples but also enables the diffusion of ions and the beneficial properties of the uniformly incorporated Ir and Rh nanoparticles.

**Figure 2:**
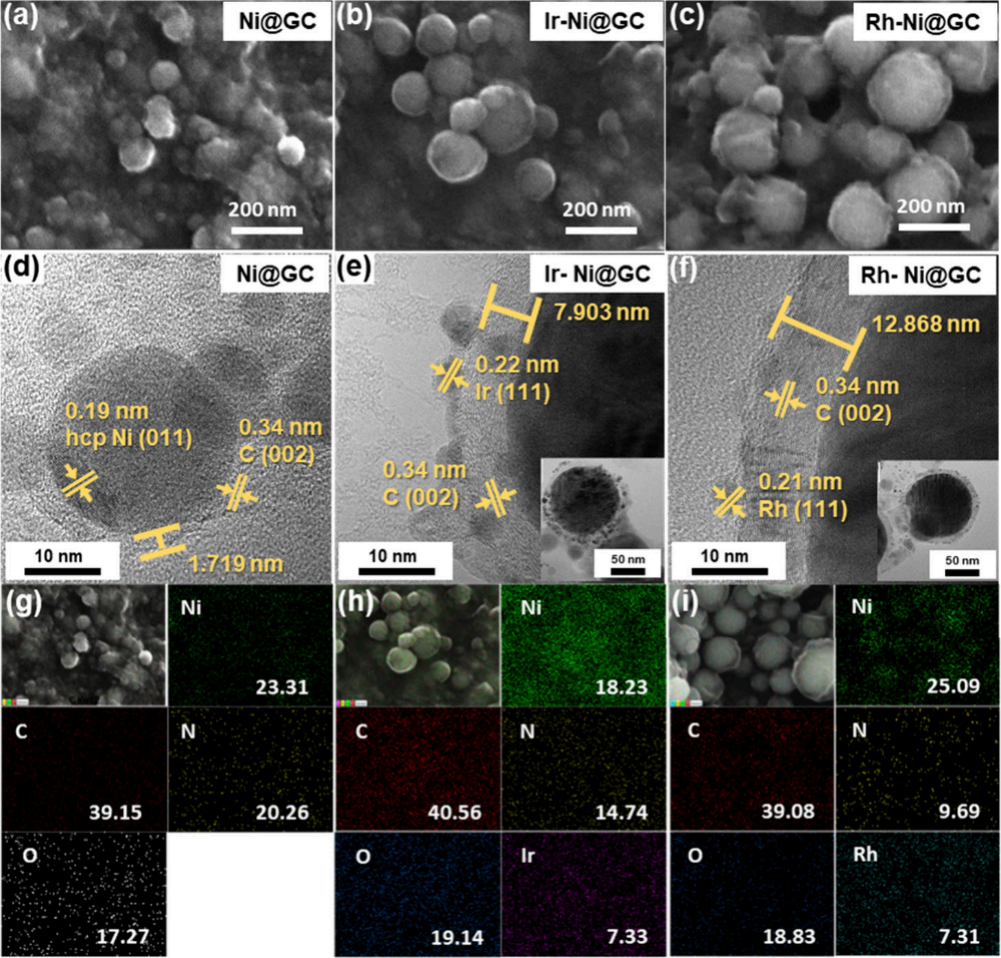
(a–c) FE-SEM images and (d–f) HR-TEM images of Ni@GC, Ir-Ni@GC, and Rh-Ni@GC materials, respectively. (g–i) EDS layered images and elemental mapping images for the Ni@GC, Ir-Ni@GC, and Rh-Ni@GC materials, respectively.

The chemical states and electronic interaction between the metal nanoparticles and GC shells with Ni nanospheres were analyzed by the X-ray photoelectron spectroscopy technique. The survey scan spectrum of the Ni@GC sample is shown in Figure 3(a), which represents the existence of Ni, C, N, and O species in the Ni@GC sample. In the high-resolution scan spectrum of Ni 2p as depicted in Figure 3(b), the high-intensity peaks observed at ∼855.8 and ∼873.6 eV can be attributed to 2p_3/2_ and 2p_1/2_ of Ni^2+^ species. Moreover, the peaks identified at ∼852.5 and ∼870.4 eV can be assigned to the zero oxidation states of Ni (Ni^0^), representing the existence of metallic Ni, and the peaks located at ∼857.2 and ∼875.1 eV can be assigned to Ni^3+^ oxidation states.^25^ Also, the broad peaks observed at ∼861.9 and ∼879.7 eV in the Ni 2p spectrum are attributed to the satellite peaks.^26,27^ The high-resolution spectrum of C 1s as shown in Figure 3(c) can be deconvoluted into four peaks, which are centered at ∼284.4, 285.3, 286.3, and 289.1 eV corresponding to the C=C, C–C, C–N/C–O, and C=O–C bonds, respectively, which demonstrates the successful formation of GC.^28^ The high-resolution O 1s spectrum of Figure S2­(a) of the Ni@GC sample reveals the defective oxygen peak at ∼532 eV, the lattice oxygen peak at ∼530.5 eV, and the adsorbed water oxygen peak at ∼533.8 eV, respectively. The dominant intensity of the defective oxygen peak suggests that the observed oxygen species likely originate from surface oxidation of nanoparticles exposed to the outer atmosphere.^29^ Furthermore, the high-resolution spectrum of N 1s as depicted in Figure 3(d) can be fitted into four peaks at ∼398.4, ∼399.7, ∼400.8, and ∼401.8 eV corresponding to the pyridinic-N, metal-N, pyrrolic-N, and graphitic-N species, respectively. The obtained N atoms in the carbon layer of the Ni@GC sample can efficiently improve the rich defect sites, enhance the electrical conductivities, and adjust the electronic structures of the material.^30,31^ Additionally, Figure 3(e, f) represents the survey scan spectra of Ir-Ni@GC and Rh-Ni@GC samples, respectively, which demonstrates the existence of respective metal species in the sample materials. Figure 3(g–j) shows the XPS spectra for Rh-Ni@GC. The observed high-resolution spectra of Ni 2p, C 1s, and N 1s for the Ir-Ni@GC (Figure S1­(a–c)) and Rh-Ni@GC (Figure 3(g–i)) samples show slight variation in the peak binding energies when compared with the Ni 2p, C 1s, and N 1s spectra for the Ni@GC sample (Figure 3(b–d)).

**Figure 3:**
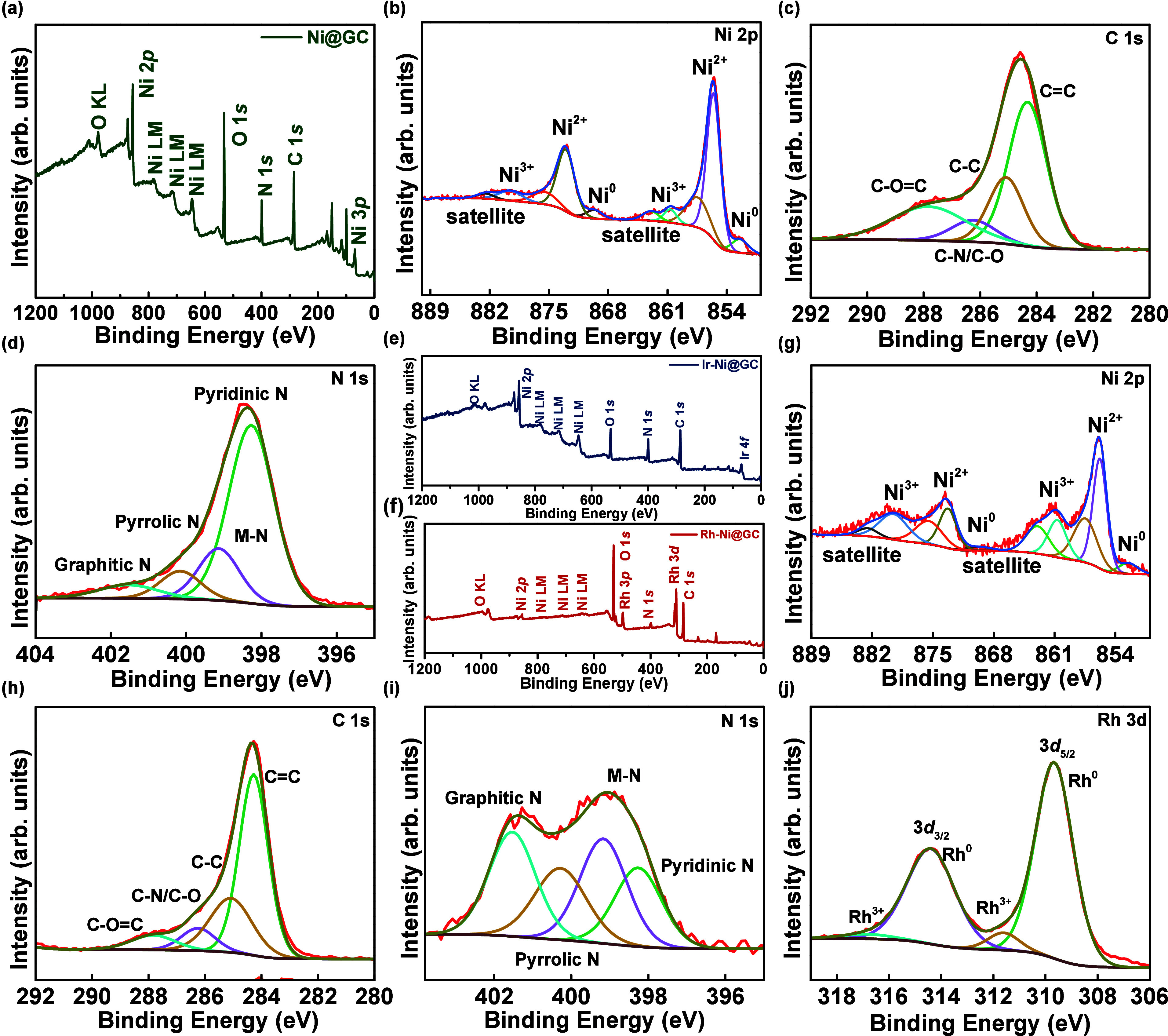
(a) Survey spectrum of Ni@GC and core-level XPS spectra of (b) Ni 2p, (c) C 1s, and (d) N 1s for Ni@GC. Survey spectra of (e) Ir-Ni@GC and (f) Rh-Ni@GC samples and core-level XPS spectra of (g) Ni 2p, (h) C 1s, (i) N 1s, and (j) Rh 3d for Rh-Ni@GC.

The high-resolution spectra of the asymmetrical-state O 1s for the Ni@GC, Ir-Ni@GC, and Rh-Ni@GC materials are presented in Figure S2­(a–c), respectively, which indicates the slight variation in the peak shift when compared with the Ni@GC material (Figure S2­(a)). The high-resolution spectra of N 1s from Figure 3(d and (i) and Figure S1­(c) indicate the graphitic-N peak intensities increase with an increase in the thickness of the GC shell on the Ni nanospheres. The thickness of the GC shell increases going from Ni@GC (1.719 nm) to Ir-Ni@GC (7.903 nm) and Rh-Ni@GC (12.868 nm), and thereby the surface area, the nitrogen content in the GC structure, and exposure of active sites increase, which can increase the relevant peak intensities. These results indicate a very strong interaction between the Ni metal core and GC shells, and the redistribution of charge on the coupling interfaces.^28^ The high-resolution spectrum of Ir 4*f* from Figure S1­(d) represents the 4*f*_7/2_ and 4*f*_5/2_ doublet peaks at ∼62.3 and ∼65.3 eV, respectively, corresponding to the Ir^0^ species, and the peaks located at ∼63.5 and ∼65.9 eV are related to the Ir^4+^ species.^32^ A slight shift is observed in the Ir peaks toward higher binding energies, due to the higher electronegativity of oxygen shifting Ir^0^ to the positive side of energies, as Ir^0^ changes to Ir^4+^ when connected with oxygen.^33,34^ The high-resolution spectrum of Rh 3d as depicted in Figure 3(j), 3*d*_5/2_ and 3*d*_3/2_ doublet peaks located at ∼309.5 and ∼314.3 eV, respectively, corresponds to the zero oxidation state (Rh^0^) of Rh metal (Δ_metal_ = 4.8 eV). Further, the peaks located at binding energies of ∼311.7 and ∼316.6 eV can be attributed to the Rh^3+^.^35^ The small intensity peaks of Rh^3+^ species originated from the surface oxidation of Rh^0^ species.^36,37^ These changes in peak shifts in the prepared Ir-Ni@GC (Ir 4*f*), and Rh-Ni@GC (Rh 3*d*) samples demonstrate the electron interactions between the noble metal (Ir and Rh) nanoparticles, GC shells, and Ni nanospheres.^38^ From the XPS analysis shown in Figure 3, the effect of the GC shell thickness on the electrocatalytic performance (HER) was investigated from the electronic structure viewpoint.

The electrocatalytic HER properties of the prepared samples are studied in a 0.5 M H_2_SO_4_ electrolyte using linear-sweep voltammetry (LSV) analysis. Figure 4(a) shows the LSV curves vs reversible hydrogen electrode (RHE) for the Ni@GC, Ir-Ni@GC, Rh-Ni@GC, and Pt/C samples measured at a scan rate of 10 mV s^–1^. From these LSV curves, the kinetic parameters of the materials such as onset potentials and overpotentials can be obtained. Generally, an efficient electrocatalyst exhibits a lower onset potential value, ideally close to the standard hydrogen reduction potential value (0 V vs. RHE). Overpotential reflects the extra energy required to overcome the kinetic barrier of the hydrogen evolution reaction (HER) and is commonly evaluated at a current density of 10 mA cm^–2^ in HER studies.^39,40^ The effect of the incorporation of noble metal nanoparticles (Ir and Rh) in Ni@GC on the HER performance was evaluated. We varied the concentrations of Ir and Rh in Ni@GC, to optimize the electrocatalyst performance. The performance of the Ir-Ni@GC and Rh-Ni@GC-based electrocatalysts was studied. The FESEM images, EDS layered images, and elemental mappings of the four different Ir contents of 3.15, 7.33, 9.91, and 14.15 wt % samples named Ir-Ni@GC-1, Ir-Ni@GC-2, Ir-Ni@GC-3, and Ir-Ni@GC-4, respectively, are shown in Figure S3­(a). The detailed weight ratio percentages of the elements (Ni, C, N, O, and Ir) in the sample are also shown in the respective elemental mapping images (Figure S3­(a)). The LSV curves for HER activity are shown in Figure S3­(b) for the samples with varying Ir content. Interestingly, the Ir content of the 7.34 wt % (Ir-Ni@GC-2) showed excellent HER activity compared to the remaining electrodes. Additionally, we synthesized Ni@GC with varied Rh contents of 3.80, 7.31, 9.64, and 15.53 wt %. The respective samples were labeled as Rh-Ni@GC-1, Rh-Ni@GC-2, Rh-Ni@GC-3, and Rh-Ni@GC-4 as shown in Figure S4. The FESEM image, EDS layered image, and elemental mapping images for the different Rh-content-based samples are shown in Figure S4­(a). Among the electrodes with varying amounts of Rh, the 7.31 wt % Rh-Ni@GC-2 sample exhibited excellent HER activity as shown in Figure S4­(b). These results demonstrate that optimal noble metal loading, along with the excellent interface between the nanoparticles and the GC shells, can significantly improve HER activity. Figure 4(a) reveals that the onset potentials for the Ni@GC, Ir-Ni@GC-2, Rh-Ni@GC-2, and Pt/C samples are −0.18, −0.078, −0.046, and −0.028 V vs. RHE, respectively. In addition, the overpotential values for the Rh-Ni@GC, Ir-Ni@GC, Ni@GC, and Pt/C samples at 10 and 50 mA cm^–2^ are shown in Figure 4(b). The Rh-Ni@GC sample revealed a lower overpotential of 46 mV when compared with the Ni@GC (180 mV) and Ir-Ni@GC (78 mV) samples and slightly higher overpotential than the Pt/C sample (28 mV at a current density of 10 mA cm^–2^, as shown in Figure 4(b). At a current density of 50 mA cm^–2^, the Rh-Ni@GC sample revealed a significantly lower overpotential of 113 mV when compared with the remaining Ni@GC (440 mV) and Ir-Ni@GC (175 mV) samples and slightly higher than that of the Pt/C sample (49 mV) as shown in Figure 4(b). Thus, although metallic Ni is unstable in an acidic medium, the thick GC shells on the surface of Ni nanospheres can enhance the stability of Ni species in an acidic medium. The obtained results suggest that the Rh-Ni@GC sample exhibits excellent HER electrocatalytic activity compared to the counter electrocatalyst materials, namely, Ni@GC and Ir-Ni@GC. Furthermore, the observed lower overpotentials indicate the excellent conductivity of the Rh-Ni@GC sample, as it achieves a lower overpotential at 10 mA cm^–2^ compared to the counter catalysts, with performance slightly exceeding that of the Pt/C sample, as shown in Figure 4(a). The electrochemical activity of as-prepared materials was analyzed using the Tafel polarization. It involves applying an overpotential (the difference between the actual electrode potential and the equilibrium potential) to an electrode and measuring the resulting current density. The relationship between the overpotential and the current density is then plotted on a logarithmic scale, known as a Tafel plot. A low Tafel slope value represents the faster HER reaction kinetics. The Tafel slope can be calculated using the Tafel equation (*η = b* log *j + a*, where η is the overpotential, *j* is the current density, *a* is the constant, and *b* is the Tafel slope).^41^ The linear portion of the Tafel plots of the Ni@GC, Ir-Ni@GC, Rh-Ni@GC, and Pt/C samples is shown in Figure 4(c). From Figure 4(c), the calculated Tafel slope values for the Ni@GC, Ir-Ni@GC, Rh-Ni@GC, and Pt/C samples are 172, 59, 36, and 35 mV dec^–1^, respectively. Rh-Ni@GC shows a lower Tafel slope than Ni@GC and Ir-Ni@GC and a slightly higher slope than Pt/C. Nickel is prone to oxidation and leaching, especially in acidic media. Encapsulation with a carbon shell such as graphene, carbon nanotubes, or amorphous carbon can provide a protective barrier against oxidation and dissolution. Further, the noble metals (Pt, Rh, Ir, and Ru) are naturally resistant to corrosion. Thus, they can improve the catalyst lifespan. The HER requires optimal hydrogen binding energy (HBE) to balance H adsorption/desorption. Ni alone has suboptimal HBE, but doping with noble metals (Rh and Ir) fine-tunes the binding energy for faster HER kinetics. In Rh-Ni@GC, the synergistic effect between the Ni nanospheres, Rh nanoparticles, and GC shells could control the electronic structure and boost transfer of proton.^42,43^ Table S1 represents the HER catalytic activity of the Rh-Ni@GC electrocatalyst compared with previous work.

**Figure 4:**
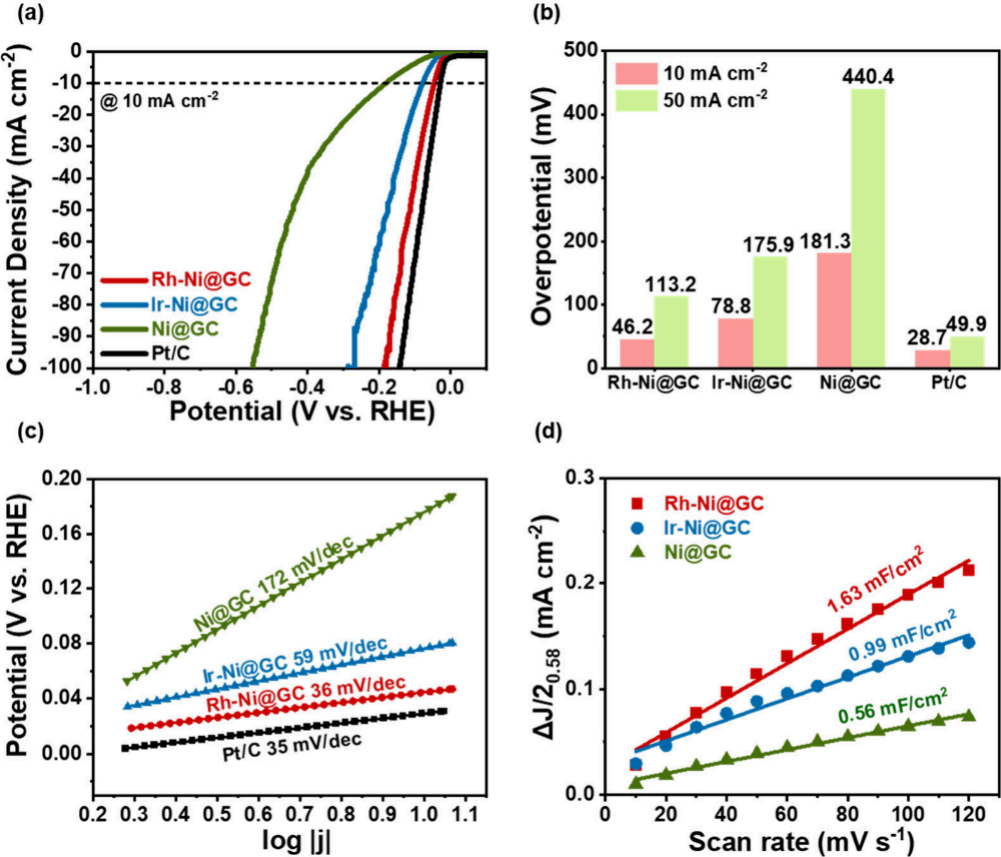
(a) Linear sweep voltammetry curves toward HER for Ni@GC, Ir-Ni@GC, Rh-Ni@GC, and Pt/C electrocatalysts in 0.5 M H_2_SO_4_. (b) Overpotential (η) values at a current density of 10 mA cm^–2^, (c) Tafel plots with corresponding Tafel slope values, and (d) double-layer capacitance (C_dl_) measurements of Ni@GC, Ir-Ni@GC, and Rh-Ni@GC, respectively.

Cyclic voltammetry (CV) analysis was conducted for the Rh-Ni@GC, Ir-Ni@GC, and Ni@GC samples at a scan rate of 50 mV s^–1^ within the potential window of 0 to 1.0 V vs. RHE as shown in Figure S5­(a–c), respectively. Rh-Ni@GC exhibited the highest number of available active sites (S_a_) among the samples, indicating superior HER activity. This enhancement can be attributed to the GC shell, which improves the charge transport between the active species and the current collector. In addition, it buffers internal volumetric expansion and suppresses structural pulverization of the material.^44^ The overall catalytic activity is strongly influenced by the enhanced active surface area, which originates from the unique nanoparticle morphology with GC shell structures. This morphology maximizes contact between the electrocatalyst surface and electrolyte by providing abundant reactive sites for HER.

The turnover frequency (TOF) values and mass activity comparison between the Ni@GC, Ir-Ni@GC, Rh-Ni@GC, and Pt/C samples across different potentials of −0.03, −0.05, −0.07, and −0.09 V are illustrated in Figure 5(a and b). From Figure 5(a), at a potential of 30 mV, the Rh-Ni@GC catalyst has a TOF value of 15.02 s^–1^, which is higher than that of the Pt/C catalyst. Also, at a potential of −0.09 V vs. RHE, the TOF value was 4.36 s^–1^, which is 2 times higher than that of the Pt/C catalyst. The Rh-Ni@GC reveals high mass activity, indicating that the modified electronic structures obtained from Rh nanoparticles, GC shell structures, and Ni nanospheres improve the intrinsic activity of HER. Moreover, Figure S8 shows that at a potential of −0.09 V vs. RHE, the mass activity of noble metals in Rh-Ni@GC is ∼2.4 times higher than that of Ir-Ni@GC and ∼1.6 times higher than that of Pt/C, respectively. Additionally, the double-layer capacitance (C_dl_) values of the electrocatalyst samples were measured using CV analysis as shown in Figure S6. Using this C_dl_ value, the electrochemically active surface area (ECSA) can be calculated. Figure S6a–c represents the CV curve profiles for the synthesized Ni@GC, Ir-Ni@GC, and Rh-Ni@GC samples, respectively. Consequently, the calculated C_dl_ values for the Rh-Ni@GC, Ir-Ni@GC, and Ni@GC samples are 0.56, 0.99, and 1.63 mF cm^–2^, respectively, as depicted in Figure 4(d). From the observed C_dl_ values, the Rh-Ni@GC sample delivered the highest C_dl_ value of 1.63 mF cm^–2^ among them. The procured highest ECSA of the Rh-Ni@GC sample demonstrates that the nanosphere morphology covered with GC shells and Rh nanoparticle incorporated structure is beneficial to exposing the number of active sites for the electrocatalytic reactions. Furthermore, the porous GC shells with Rh nanoparticles incorporated on the surface of Ni nanospheres could promote hydrogen to detach from the electrode and support quick mass transfer, thus improving the HER properties of the Rh-Ni@GC sample.^45^ The electrical conductivities of as-prepared Ni@GC, Ir-Ni@GC, Rh-Ni@GC, and Pt/C samples were analyzed by EIS analysis. Figure 5(c) demonstrates the Nyquist plots of the prepared samples at an AC amplitude of 5 V. The charge transfer resistance (R_ct_) value of the Rh-Ni@GC sample (15.27 Ω) is lower than that of the counter samples such as Ni@GC and Ir-Ni@GC samples. Remarkably, the obtained EIS results were consistent with the above LSV and Tafel plot analysis, and they showed that the Rh-Ni@GC stable catalyst exhibited better electrocatalytic properties than other materials. The obtained better performance can be ascribed to the small size, uniform distribution, and maximum intrinsic activity of Rh nanoparticles.^37^ This suggests that the Ni nanospheres were well protected by the GC shells and uniformly incorporated with Rh nanoparticles, thereby providing excellent electrocatalytic properties toward HER activity.^38^ It has been reported that the antibonding states between the Rh, GC network and adsorbed intermediates are less occupied, thereby lowering the binding strength and enhancing HER activity.^46^ From the corresponding Bode plot of Figure S9­(a), the Rh-Ni@GC sample exhibits a distinct decrease and change in phase angles when compared with the Ni@GC and Ir-Ni@GC samples and a slight increase and change in phase angle with the Pt/C sample. Moreover, the Nyquist plots and respective Bode plots of the Rh-Ni@GC sample at various applied HER overpotentials are shown in Figure S10­(a and b), respectively. This demonstrates the fast transfer of electrons in the collaborative Rh-Ni@GC sample interface for the conversion of surface-adsorbed OH ions and intermediates.^47,48^ The long-term stability test is an indispensable evaluation concept for HER performance. The stability test of the Rh-Ni@GC sample was investigated by chronoamperometry analysis over 24 h as shown in Figure 5(d). The current vs time graph was obtained and it demonstrated that the current response remains the same over 24 h of electrochemical operation, which reveals the excellent stability of the Rh-Ni@GC catalyst. These results suggest that our strategy provides a cost-effective route for the scalable synthesis of electrocatalysts with controlled compositions and structures for the hydrogen evolution reaction.

**Figure 5:**
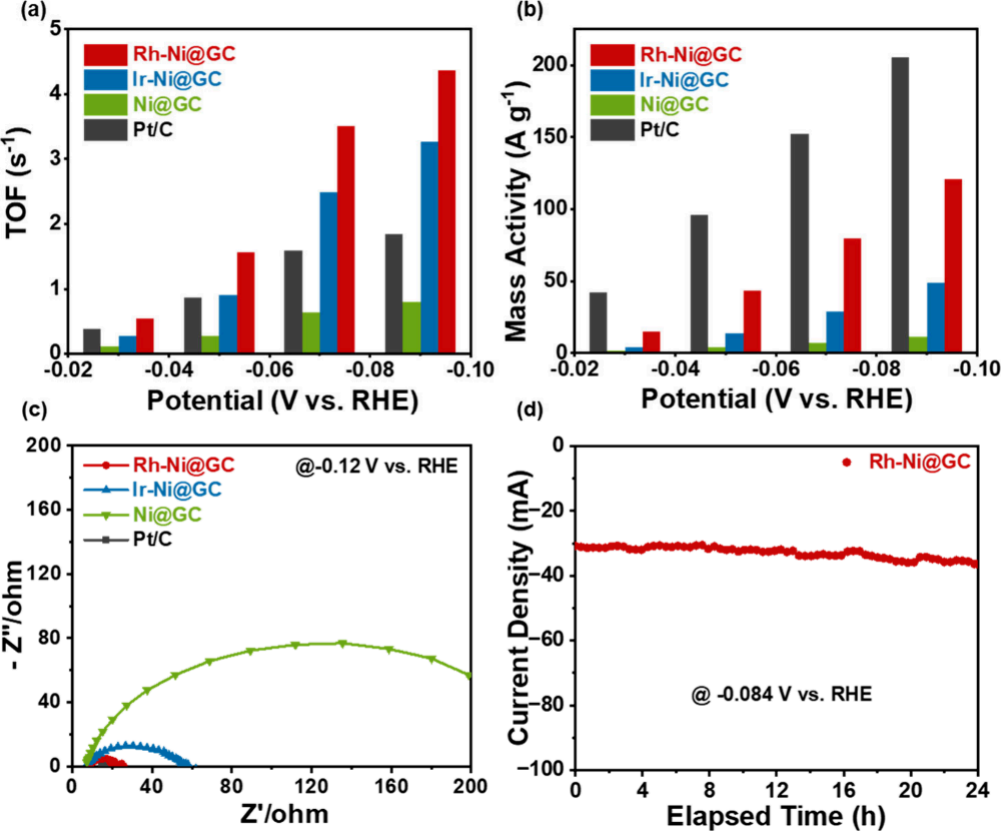
(a) Turnover frequency values, (b) mass activity at different overpotentials, and (c) Nyquist impedance plots for the Ni@GC, Ir-Ni@GC, Rh-Ni@GC, and Pt/C samples. (d) Stability test of Rh-Ni@GC in 0.5 M H_2_SO_4_ at −0.084 V vs RHE.

Herein, the Rh-Ni@GC electrocatalyst synthesized using the PLAL method showed excellent catalytic activity and stability for the HER. Also, while the pulsed laser method provides a green, surfactant-free synthesis route with precise control over morphology and particle distribution, the scalability of this method is a challenge.^49^ Therefore, developing laser-compatible scale-up strategies is crucial. Moreover, Rh is an expensive noble metal and this high cost hinders large-scale adoption.^50^ To overcome this issue, our study demonstrates the ultralow Rh loading with maximized utilization via atomic dispersion, which could be further optimized in future studies. The practical implementation also demands long-term operational durability under industrial conditions.^51^ Therefore, additional tests such as large-area electrode fabrication and stack integration are essential next steps to ensure compatibility with hydrogen production systems. Future studies would include (1) development of metal nanoparticle combinations, such as Ni, Co, Cu, Fe, etc., for cost optimization and integration of the electrocatalyst material into various energy systems and (2) testing the long-term performance at different pH and temperature conditions to evaluate industrial viability. The above-mentioned economic considerations and practical implementation strategies could be beneficial toward sustainable hydrogen production technologies.

## 4 Conclusions

In this work, we successfully prepared Ir and Rh nanoparticles uniformly incorporated on the surface of graphitic carbon-covered Ni nanospheres using the innovative and facile two-step pulsed laser technique. The XPS results highlighted the synergistic interactions between Rh and Ni, suggesting that their optimized electronic configuration contributes to a superior catalytic performance. These findings underscore the structural and electronic advantages of the synthesized material, demonstrating its potential for advanced electrochemical applications. The optimal loading of the Rh-Ni@GC revealed excellent electrocatalytic activities toward HER than those of Ni@GC and Ir-Ni@GC catalysts. Moreover, when compared with the commercial Pt/C catalyst, the Rh-Ni@GC catalyst exhibited a comparable overpotential value (46 mV) at a current density of 10 mA cm^–2^ and Tafel slope value (36 mV dec^–1^) in 0.5 M H_2_SO_4_ acidic electrolyte. The prepared Rh-Ni@GC revealed better impedance spectroscopy properties and excellent stability compared to its counterparts. Additionally, the superior electrocatalytic performance can be attributed to the synergistic effect among the Rh nanoparticles, controllable graphitic carbon layers, and the Ni nanosphere interface. This results in strong electronic interactions, abundant active sites, favorable reaction kinetics, and enhanced electrical conductivity. Finally, this work provides an avenue to synthesize efficient noble metal nanoparticles incorporated on the surface of the metallic core covered by graphitic carbon layers for improved electrocatalytic performances.

## Supplementary Material



## Notes

High-resolution XPS spectra of Ni 2p, C 1s, N 1s, Ir 4f, and O 1s, SEM and SEM-EDS analyses, LSV curves showing the effect of Ir and Rh content, CV profiles in 0.5 M H_2_SO_4_, CV curves in the non-Faradaic region at various scan rates, TOF and mass activity comparisons across catalysts, noble metal mass activity at different potentials, Bode and Nyquist plots, EIS at different HER overpotentials, and comparison table of HER performance with reported Rh-based catalysts (PDF)

## References

[1] Ji M., Wang J. (2021). Review and comparison of various hydrogen production methods based
on costs and life cycle impact assessment indicators. Int. J. Hydrogen Energy.

[2] Elaouzy Y., El Fadar A. (2024). Water-energy-carbon-cost
nexus in hydrogen production,
storage, transportation and utilization. Int.
J. Hydrogen Energy.

[3] Lu J., Xiong T., Zhou W., Yang L., Tang Z., Chen S. (2016). Metal nickel foam as
an efficient and stable electrode for hydrogen
evolution reaction in acidic electrolyte under reasonable overpotentials. ACS Appl. Mater. Interfaces.

[4] Ledendecker M., Mondschein J. S., Kasian O., Geiger S., Göhl D., Schalenbach M., Zeradjanin A., Cherevko S., Schaak R. E., Mayrhofer K. (2017). Stability and activity of non-noble-metal-based catalysts
toward the hydrogen evolution reaction. Angew.
Chem., Int. Ed..

[5] Wang J., Yue X., Yang Y., Sirisomboonchai S., Wang P., Ma X., Abudula A., Guan G. (2020). Earth-abundant transition-metal-based
bifunctional catalysts for overall electrochemical water splitting:
A review. J. Alloys Compd..

[6] Yoo J. M., Shin H., Chung D. Y., Sung Y.-E. (2022). Carbon
shell on
active nanocatalyst for stable electrocatalysis. Accounts of chemical research.

[7] Hu K., Ohto T., Chen L., Han J., Wakisaka M., Nagata Y., Fujita J.-i., Ito Y. (2018). Graphene layer
encapsulation
of non-noble metal nanoparticles as acid-stable hydrogen evolution
catalysts. ACS Energy Letters.

[8] Ding R., Yan T., Wang Y., Long Y., Fan G. (2021). Carbon nanopore and
anchoring site-assisted general construction of encapsulated metal
(Rh, Ru, Ir) nanoclusters for highly efficient hydrogen evolution
in pH-universal electrolytes and natural seawater. Green Chem..

[9] Yu J., Dai Y., Wu X., Zhang Z., He Q., Cheng C., Wu Z., Shao Z., Ni M. (2021). Ultrafine ruthenium-iridium alloy
nanoparticles well-dispersed on N-rich carbon frameworks as efficient
hydrogen-generation electrocatalysts. Chemical
Engineering Journal.

[10] Yang G. W. (2007). Laser ablation
in liquids: Applications in the synthesis of nanocrystals. Prog. Mater. Sci..

[11] Zhang D., Gökce B., Barcikowski S. (2017). Laser Synthesis and Processing of
Colloids: Fundamentals and Applications. Chem.
Rev..

[12] Forsythe R. C., Cox C. P., Wilsey M. K., Muller A. M. (2021). Pulsed
laser in
liquids made nanomaterials for catalysis. Chem.
Rev..

[13] Zeng H., Du X. W., Singh S. C., Kulinich S. A., Yang S., He J., Cai W. (2012). Nanomaterials via laser
ablation/irradiation in liquid:
a review. Adv. Funct. Mater..

[14] Zhang D., Li Z., Sugioka K. (2021). Laser ablation
in liquids for nanomaterial synthesis:
Diversities of targets and liquids. Journal
of Physics: Photonics.

[15] Theerthagiri J., Karuppasamy K., Lee S. J., Shwetharani R., Kim H.-S., Pasha S. K., Ashokkumar M., Choi M. Y. (2022). Fundamentals and comprehensive insights on pulsed laser
synthesis of advanced materials for diverse photo-and electrocatalytic
applications. Light: Science & Applications.

[16] Jiang Y., Su J., Yang Y., Jia Y., Chen Q., Xie Z., Zheng L. (2016). A facile surfactant-free
synthesis of Rh flower-like nanostructures
constructed from ultrathin nanosheets and their enhanced catalytic
properties. Nano Research.

[17] Lee S. J., Theerthagiri J., Choi M. Y. (2022). Time-resolved dynamics of laser-induced
cavitation bubbles during production of Ni nanoparticles via pulsed
laser ablation in different solvents and their electrocatalytic activity
for determination of toxic nitroaromatics. Chemical
Engineering Journal.

[18] Zou Q., Dai L., Li Y., Wang Z., Yu Q., Luo Y., Luo W. (2023). Microstructure
of onion-like carbon fabricated in vacuum by annealing
ND. Diamond Relat. Mater..

[19] Kim J., Begildayeva T., Theerthagiri J., Moon C. J., Min A., Lee S. J., Kim G.-A., Choi M. Y. (2023). Manifolding active
sites and in situ/operando electrochemical-Raman spectroscopic studies
of single-metal nanoparticle-decorated CuO nanorods in furfural biomass
valorization to H2 and 2-furoic acid. Journal
of Energy Chemistry.

[20] Caporali M., Serrano-Ruiz M., Telesio F., Heun S., Nicotra G., Spinella C., Peruzzini M. (2017). Decoration
of exfoliated black phosphorus
with nickel nanoparticles and its application in catalysis. Chem. Commun..

[21] Ferrari A. C., Robertson J. (2000). Interpretation
of Raman spectra of disordered and amorphous
carbon. Phys. Rev. B.

[22] Jung H. J., Choi M. Y. (2018). One-pot synthesis
of graphitic and nitrogen-doped graphitic
layers on nickel nanoparticles produced by pulsed laser ablation in
liquid: Solvent as the carbon and nitrogen source. Appl. Surf. Sci..

[23] Shen Y., Zhou Y., Wang D., Wu X., Li J., Xi J. (2018). Nickel-copper alloy encapsulated in graphitic carbon
shells as electrocatalysts
for hydrogen evolution reaction. Adv. Energy
Mater..

[24] Jung H. J., Choi M. Y. (2014). Specific solvent produces specific phase Ni nanoparticles:
a pulsed laser ablation in solvents. J. Phys.
Chem. C.

[25] Li T., Luo G., Liu K., Li X., Sun D., Xu L., Li Y., Tang Y. (2018). Encapsulation of Ni3Fe nanoparticles in N-doped carbon
nanotube-grafted carbon nanofibers as high-efficiency hydrogen evolution
electrocatalysts. Adv. Funct. Mater..

[26] Li D., Liao L., Zhou H., Zhao Y., Cai F., Zeng J., Liu F., Wu H., Tang D., Yu F. (2021). Highly active non-noble electrocatalyst
from Co2P/Ni2P nanohybrids
for pH-universal hydrogen evolution reaction. Materials today physics.

[27] Cheng W., Lu X. F., Luan D., Lou X. W. (2020). NiMn-Based bimetal-organic
framework nanosheets supported on multi-channel carbon fibers for
efficient oxygen electrocatalysis. Angew. Chem.,
Int. Ed..

[28] Li Z., Wu X., Jiang X., Shen B., Teng Z., Sun D., Fu G., Tang Y. (2022). Surface carbon layer controllable Ni3Fe particles confined
in hierarchical N-doped carbon framework boosting oxygen evolution
reaction. Advanced Powder Materials.

[29] Prieto P., Nistor V., Nouneh K., Oyama M., Abd-Lefdil M., Díaz R. (2012). XPS study
of silver, nickel and bimetallic silver-nickel
nanoparticles prepared by seed-mediated growth. Appl. Surf. Sci..

[30] Wang S., Wang H., Huang C., Ye P., Luo X., Ning J., Zhong Y., Hu Y. (2021). Trifunctional
electrocatalyst
of N-doped graphitic carbon nanosheets encapsulated with CoFe alloy
nanocrystals: The key roles of bimetal components and high-content
graphitic-N. Applied Catalysis B: Environmental.

[31] Wang Q., Ji Y., Lei Y., Wang Y., Wang Y., Li Y., Wang S. (2018). Pyridinic-N-dominated
doped defective graphene as a superior oxygen
electrocatalyst for ultrahigh-energy-density Zn-air batteries. ACS Energy Letters.

[32] Wang H., Ming M., Hu M., Xu C., Wang Y., Zhang Y., Gao D., Bi J., Fan G., Hu J.-S. (2018). Size and electronic modulation of iridium nanoparticles
on nitrogen-functionalized
carbon toward advanced electrocatalysts for alkaline water splitting. ACS Appl. Mater. Interfaces.

[33] Da
Silva L., Alves V., De Castro S., Boodts J. (2000). XPS study of the state of iridium, platinum, titanium
and oxygen in thermally formed IrO2+ TiO2+ PtOX films. Colloids Surf., A.

[34] Labou D., Slavcheva E., Schnakenberg U., Neophytides S. (2008). Performance
of laboratory polymer electrolyte membrane hydrogen generator with
sputtered iridium oxide anode. J. Power Sources.

[35] Golvano-Escobal I., Surinach S., Baró M. D., Pane S., Sort J., Pellicer E. (2016). Electrodeposition of sizeable and compositionally tunable
rhodium-iron nanoparticles and their activity toward hydrogen evolution
reaction. Electrochim. Acta.

[36] Guo Y., Wang Y., Huang Z., Tong X., Yang N. (2022). Size effect
of Rhodium nanoparticles supported on carbon black on the performance
of hydrogen evolution reaction. Carbon.

[37] Wang Q., Ming M., Niu S., Zhang Y., Fan G., Hu J. S. (2018). Scalable solid-state
synthesis of highly dispersed uncapped metal
(Rh, Ru, Ir) nanoparticles for efficient hydrogen evolution. Adv. Energy Mater..

[38] Jiang B., Huang A., Wang T., Shao Q., Zhu W., Liao F., Cheng Y., Shao M. (2020). Rhodium/graphitic-carbon-nitride
composite electrocatalyst facilitates efficient hydrogen evolution
in acidic and alkaline electrolytes. J. Colloid
Interface Sci..

[39] Li J., Zheng G. (2017). One-dimensional earth-abundant nanomaterials for water-splitting
electrocatalysts. Advanced Science.

[40] Tang Y. J., Wang Y., Wang X. L., Li S. L., Huang W., Dong L. Z., Liu C. H., Li Y. F., Lan Y. Q. (2016). Molybdenum
disulfide/nitrogen-doped reduced graphene oxide nanocomposite with
enlarged interlayer spacing for electrocatalytic hydrogen evolution. Adv. Energy Mater..

[41] Oh Y., Theerthagiri J., Min A., Moon C. J., Yu Y., Choi M. Y. (2024). Pulsed laser interference patterning of transition-metal
carbides for stable alkaline water electrolysis kinetics. Carbon Energy.

[42] Deng J., Ren P., Deng D., Yu L., Yang F., Bao X. (2014). Highly active
and durable non-precious-metal catalysts encapsulated in carbon nanotubes
for hydrogen evolution reaction. Energy Environ.
Sci..

[43] Yang Y., Lun Z., Xia G., Zheng F., He M., Chen Q. (2015). Non-precious
alloy encapsulated in nitrogen-doped graphene layers derived from
MOFs as an active and durable hydrogen evolution reaction catalyst. Energy Environ. Sci..

[44] Li R., Wang Y., Zhou C., Wang C., Ba X., Li Y., Huang X., Liu J. (2015). Carbon-stabilized high-capacity ferroferric
oxide nanorod array for flexible solid-state alkaline battery-supercapacitor
hybrid device with high environmental suitability. Adv. Funct. Mater..

[45] Shang L., Zhao Y., Kong X.-Y., Shi R., Waterhouse G. I., Wen L., Zhang T. (2020). Underwater
superaerophobic Ni nanoparticle-decorated
nickel-molybdenum nitride nanowire arrays for hydrogen evolution in
neutral media. Nano Energy.

[46] Ruban A., Hammer B., Stoltze P., Skriver H. L., Nørskov J. K. (1997). Surface
electronic structure and reactivity of transition and noble metals. J. Mol. Catal. A: Chem..

[47] Zhou D., Wang S., Jia Y., Xiong X., Yang H., Liu S., Tang J., Zhang J., Liu D., Zheng L. (2019). NiFe hydroxide
lattice tensile strain: enhancement of adsorption
of oxygenated intermediates for efficient water oxidation catalysis. Angew. Chem., Int. Ed..

[48] Maruthapandian V., Mathankumar M., Saraswathy V., Subramanian B., Muralidharan S. (2017). Study of the
oxygen evolution reaction catalytic behavior
of Co x Ni1-x Fe2O4 in alkaline medium. ACS
Appl. Mater. Interfaces.

[49] Iacono V., Mirabella S., Ruffino F. (2023). Efficient Oxygen Evolution Reaction
Catalyzed by Ni/NiO Nanoparticles Produced by Pulsed Laser Ablation
in Liquid Environment. physica status solidi
(b).

[50] Begildayeva T., Chinnadurai D., Lee S. J., Yu Y., Song J. K., Choi M. Y. (2022). Implementation of novel pulsed laser ablation strategy
to control the morphological growth and enrich the electrochemically
active sites of multifunctional Ni-CuO electrocatalyst. J. Alloys Compd..

[51] Santos A. L., Cebola M.-J., Santos D. M. (2021). Towards the hydrogen economy—A
review of the parameters that influence the efficiency of alkaline
water electrolyzers. Energies.

