# Supplementary material for: Rhodium Nanoparticle-Supported Graphitic Carbon-Encapsulated Nickel Metal Core Electrocatalyst via Pulsed Laser Ablation for Hydrogen Evolution Reaction
Source: ACS Appl Mater Interfaces. 2025 Jul 29;17(31):44402–10. doi: 10.1021/acsami.5c07778 (PMC12332833; doi:10.1021/acsami.5c07778)
Supplement: Supplementary file 1 [file am5c07778_si_001.pdf]

Supporting Information

Rhodium Nanoparticles Supported Graphitic Carbon Encapsulated Nickel Metal Core Electrocatalyst via Pulsed Laser Ablation for Hydrogen Evolution Reaction

Yewon Oh [b], *B. N. Vamsi Krishna* [a], *Hyeon Jin Jung** [c], *Anju Toor** [b], *Seung Jun Lee** [a]

[a] Department of IT and Energy Convergence (Brain Korea 21 FOUR), Korea National University of Transportation, Chungju 27469, South Korea.

[b] School of Materials Science and Engineering, Georgia Institute of Technology, Atlanta, Georgia 30332, USA.

[c] Nano Convergence Materials Center, Emerging Materials R&D Division, Korea Institute of Ceramic Engineering and Technology (KICET), Jinju 52851, South Korea.

*Correspondence Email address: *sjlee@ut.ac.kr, anju@gatech.edu*, *laser02hj@kicet.re.kr*

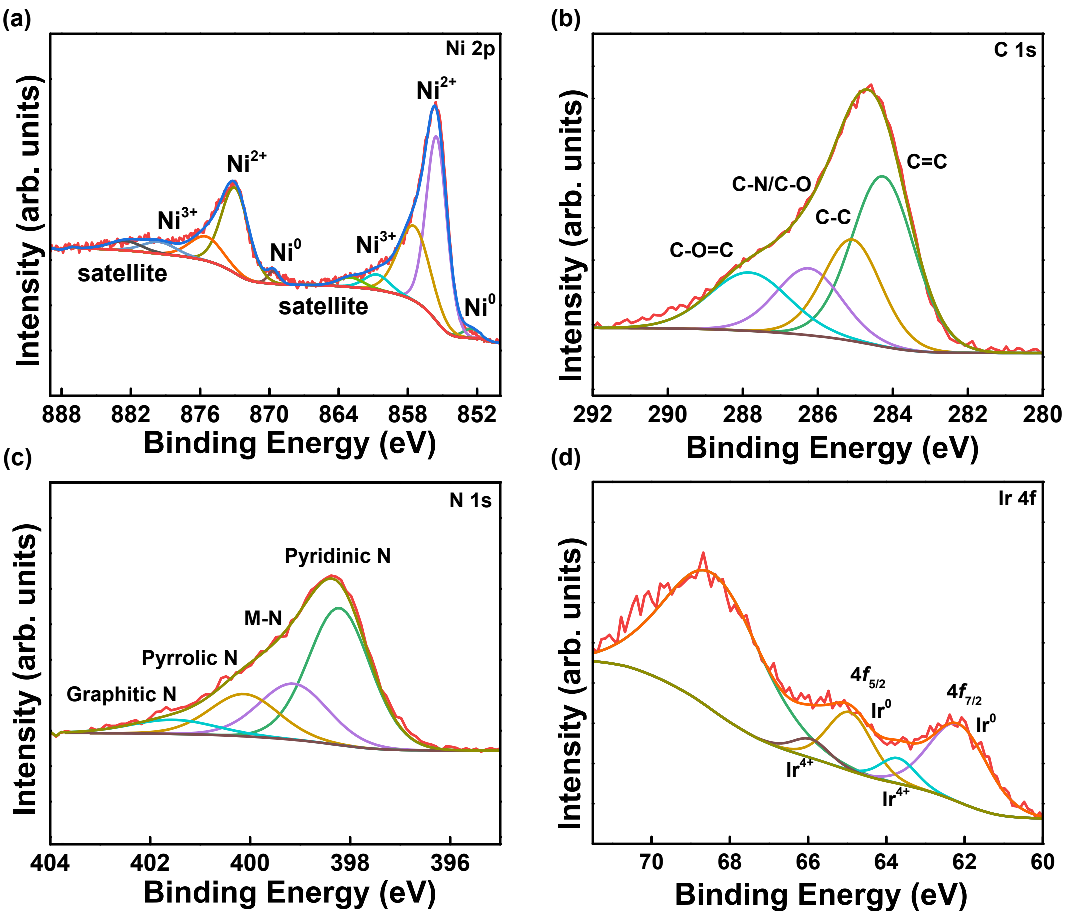

Figure S1. High-resolution XPS spectra of Ir-Ni@GC sample; (a) Ni 2p, (b) C 1s, (c) N 1s, and (d) Ir 4f.

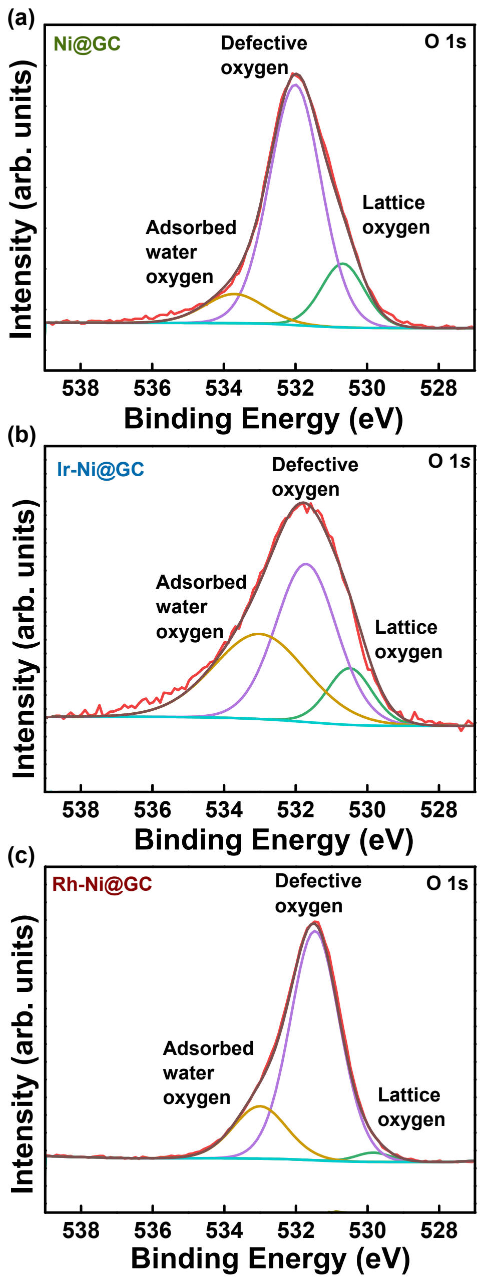

Figure S2. XPS O 1s core spectrum for (a) Ni@GC, (b) Ir-Ni@Gc, and (c) Rh-Ni@GC.

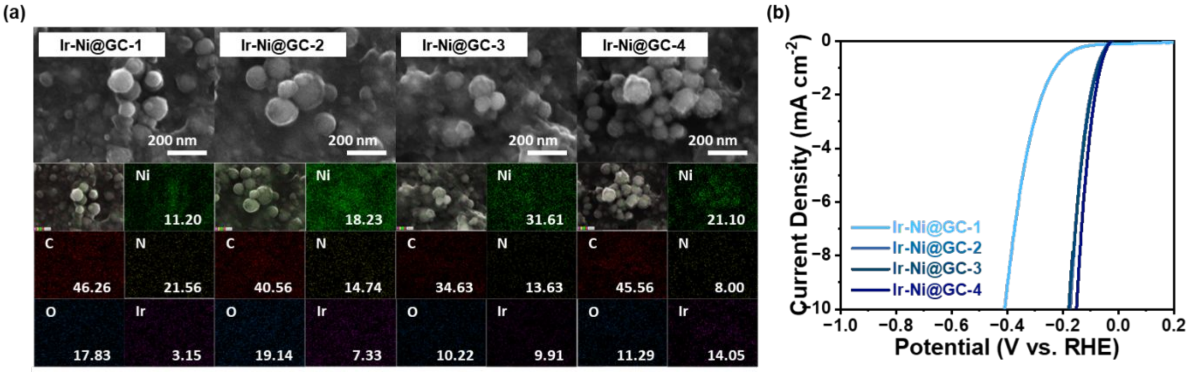

Figure S3. (a) SEM and SEM-EDS analyses showing variations with Ir content, and (b) LSV curves comparing HER activity at different Ir contents.

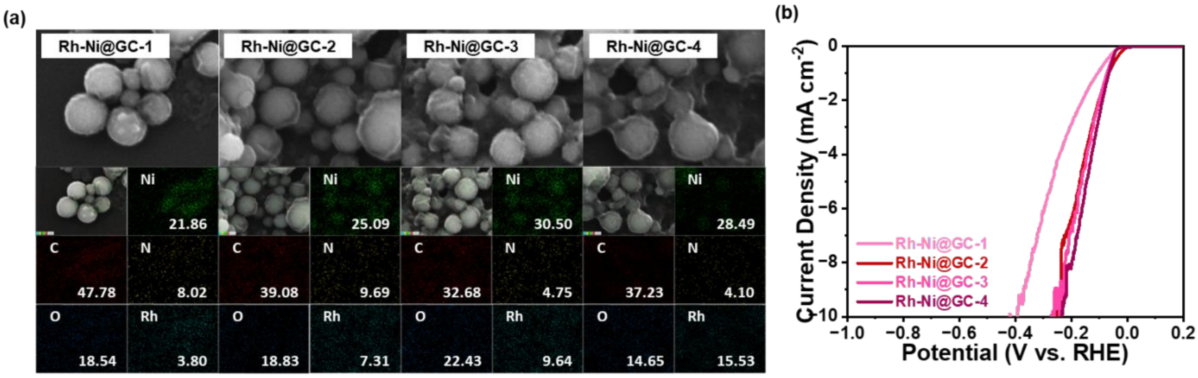

Figure S4. (a) SEM and SEM-EDS analyses showing variations with Rh content, and (b) LSV curves comparing HER activity at different Rh contents.

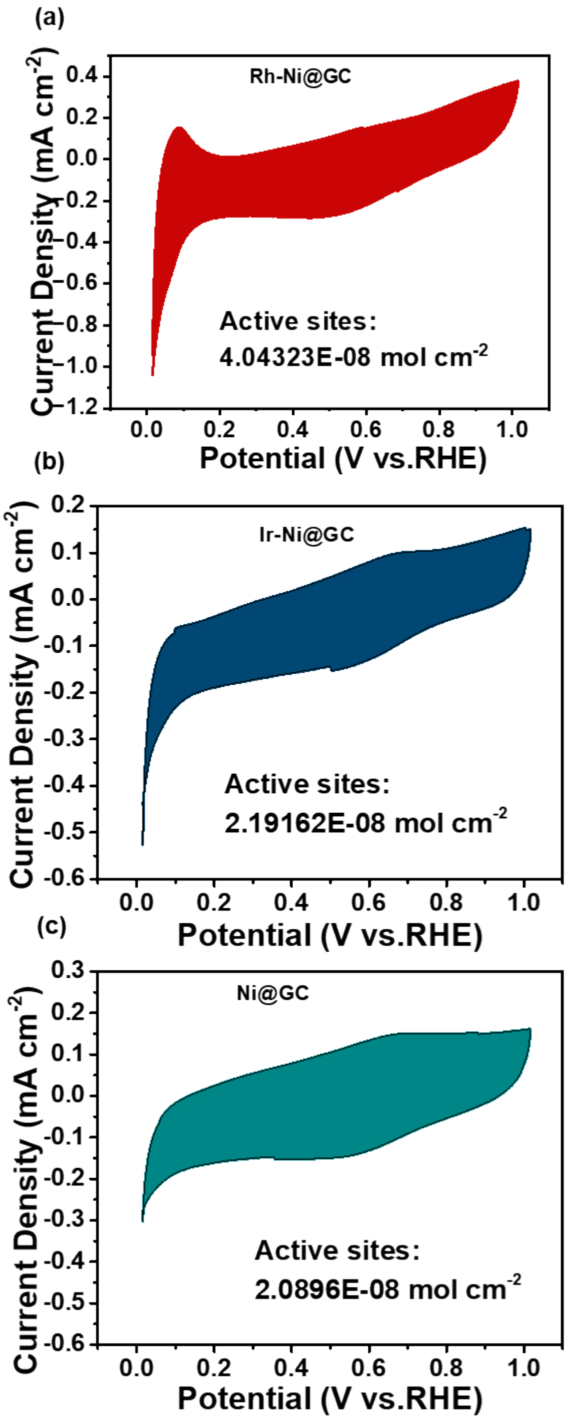

Figure S5. CV profiles of (a) Rh-Ni@GC, (b) Ir-Ni@GC, and (c) Ni@GC recorded in 0.5 M H_2SO_4 within the potential window of 0 to 1 V vs. RHE at a scan rate of 50 mV/s.

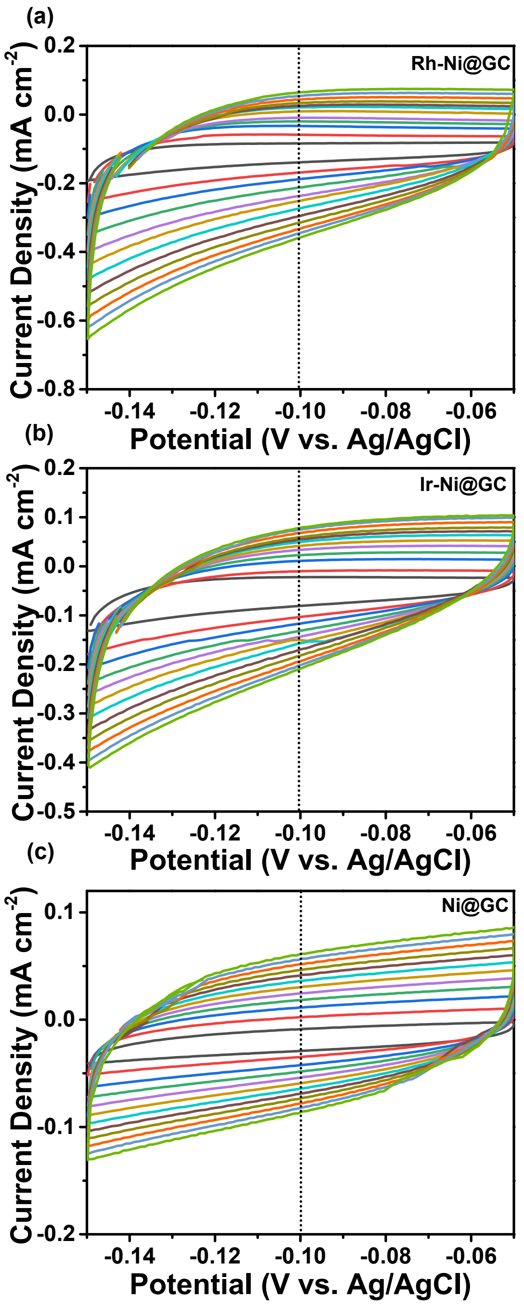

Figure S6. (a-c) CV curves of Rh-Ni@GC, Ir-Ni@GC, and Ni@GC samples measured in the non-Faradaic region in 0.5 M H_2SO_4 at scan rates ranging from 10 to 120 mV/s.

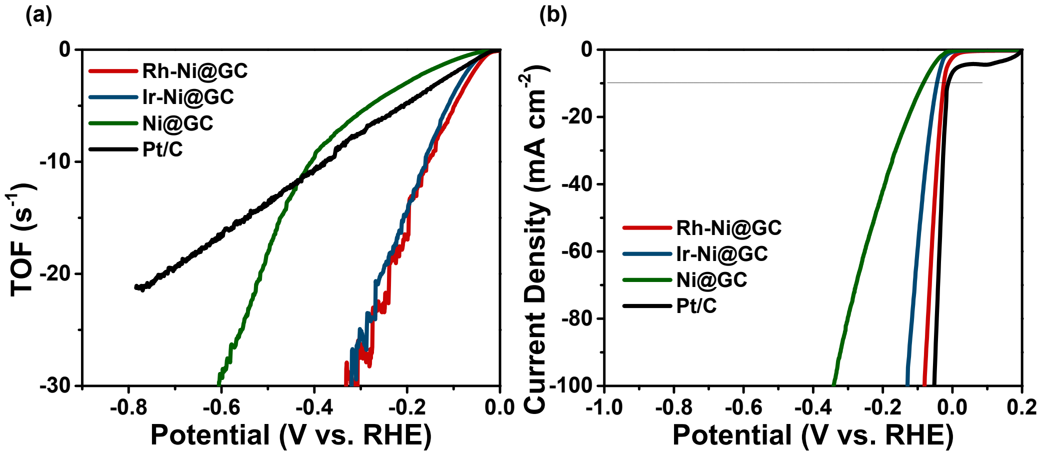

Figure S7. (a) TOF value and (b) mass activities assessed over a wide HER potential range for Pt/C, Ni@GC, Ir-Ni@GC, and Rh-Ni@GC samples.

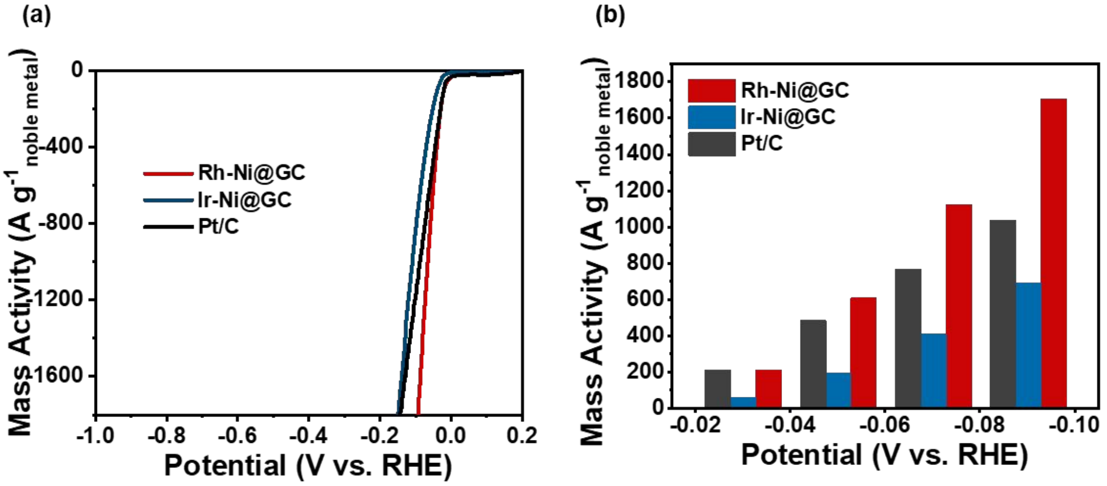

Figure S8. (a) Mass activities of noble metals and (b) a comparison of the mass activity of noble metals in Pt/C, Ir-Ni@GC, and Rh-Ni@GC samples at different potentials.

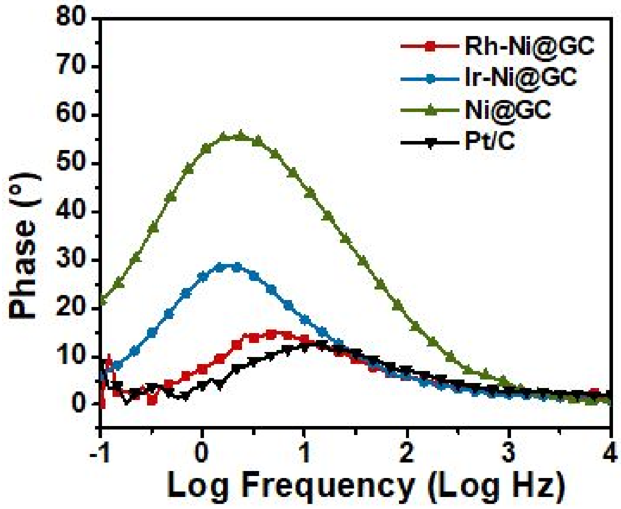

Figure S9. Bode plots of Pt/C, Ni@GC, Ir-Ni@GC, and Rh-Ni@GC samples.

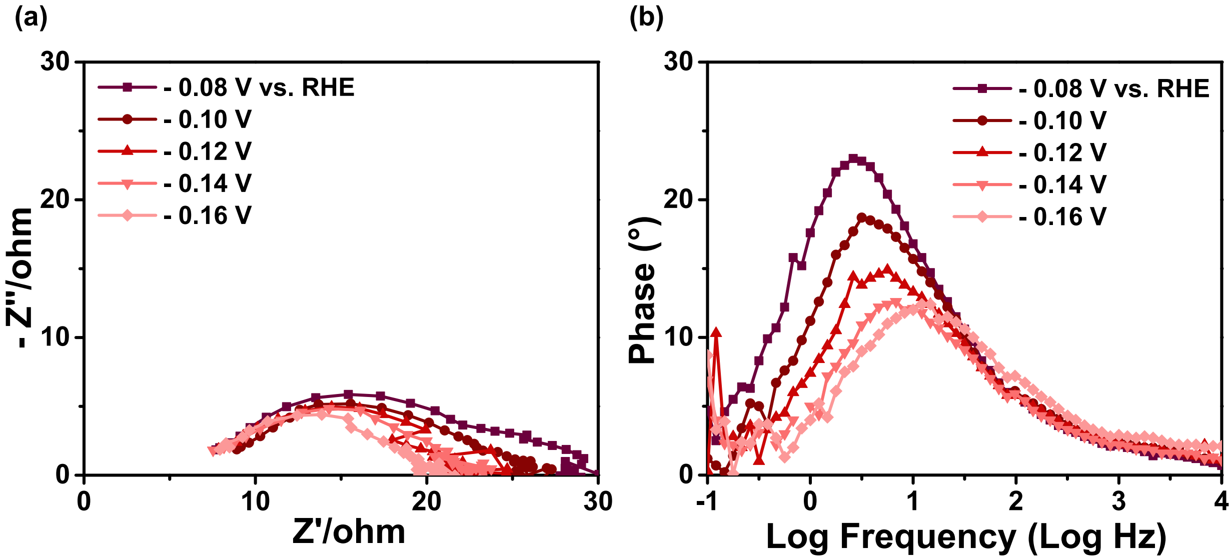

Figure S10. (a) Nyquist impedance and (b) Bode plots of the Rh-Ni@GC sample recorded at various applied HER overpotentials.

Table S1. The HER performance of an Rh-Ni@GC catalyst previously reported materials based on rhodium material.

No.	Catalysts	Overpotential (mV) @ 10 mA cm^{-2}	Tafel slope (mV dec^{-1})	Electrolyte	Ref.
1	Rh/Ni@NCNTs	45	37.2	0.5 M H_2SO_4	[1]
2	Rh/SiQDs/CQD-3	36	26	0.5 M H_2SO_4	[2]
3	RhNi alloy	40	54	0.5 M H_2SO_4	[3]
4	W-Rh/C	57	50	0.1 M H_2SO_4	[4]
5	rGO/CoP-Rh-2.5	43	72	0.5 M H_2SO_4	[5]
6	Rh/GNPs	76	42	0.5 M H_2SO_4	[6]
**7**	**Rh-Ni@GC**	**46**	**36**	**0.5 M H_2SO_4**	**This work**

Reference

(1) Wang, Q.; Xu, B.; Xu, C.; Wang, Y.; Zhang, Y.; Wu, J.; Fan, G. Ultrasmall Rh nanoparticles decorated on carbon nanotubes with encapsulated Ni nanoparticles as excellent and pH-universal electrocatalysts for hydrogen evolution reaction. *Applied Surface Science* **2019**, *495*, 143569.

(2) Dang, Q.; Liao, F.; Sun, Y.; Zhang, S.; Huang, H.; Shen, W.; Kang, Z.; Shi, Y.; Shao, M. Rhodium/silicon quantum dot/carbon quantum dot composites as highly efficient electrocatalysts for hydrogen evolution reaction with Pt-like performance. *Electrochimica Acta* **2019**, *299*, 828-834.

(3) Ehsan, M. A.; Aftab, F.; Younas, M.; Mansoor, M. A.; Ahmed, S. Graphite sheet-supported bimetallic RhNi thin film alloys for enhanced and durable hydrogen evolution in acidic environments. *International Journal of Hydrogen Energy* **2024**, *69*, 411-420.

(4) Volpato, G. A.; Arboleda, D. M.; Brandiele, R.; Carraro, F.; Sartori, G. B.; Cardelli, A.; Badocco, D.; Pastore, P.; Agnoli, S.; Durante, C. Clean rhodium nanoparticles prepared by laser ablation in liquid for high performance electrocatalysis of the hydrogen evolution reaction. *Nanoscale Advances* **2019**, *1* (11), 4296-4300.

(5) Zheng, H.; Huang, X.; Gao, H.; Dong, W.; Lu, G.; Chen, X.; Wang, G. Decorating cobalt phosphide and rhodium on reduced graphene oxide for high-efficiency hydrogen evolution reaction. *Journal of Energy Chemistry* **2019**, *34*, 72-79.

(6) Golubović, J.; Rakočević, L.; Latas, N.; Varničić, M.; Rajić, V.; Štrbac, S. Enhanced hydrogen evolution catalysis on Rh nanoparticles with low loading on graphene nanoplatelets. *Applied Surface Science* **2024**, *672*, 160805.
